# Efficacy and safety of pressurized intraperitoneal aerosol chemotherapy (PIPAC) in ovarian cancer: a systematic review of current evidence

**DOI:** 10.1007/s00404-024-07586-z

**Published:** 2024-06-15

**Authors:** Matteo Pavone, Floriane Jochum, Lise Lecointre, Nicolò Bizzarri, Cristina Taliento, Stefano Restaino, Giuseppe Vizzielli, Anna Fagotti, Giovanni Scambia, Denis Querleu, Cherif Akladios

**Affiliations:** 1 Institute of Image-Guided Surgery, IHU Strasbourg, 1 place de l’Hôpital, 67091 Strasbourg, France; 2 UOC Ginecologia Oncologica, Dipartimento di Scienze per la salute della Donna e del Bambino e di Sanità Pubblica, Fondazione Policlinico Universitario A. Gemelli, IRCCS, Rome, Italy; 3 Department of Gynecologic Surgery, University Hospitals of Strasbourg, Strasbourg, France; 4 UMR 7357, Laboratoire Des Sciences de l’Ingénieur, de l’Informatique et de l’Imagerie, ICube, Université de Strasbourg, 67000 Strasbourg, France; 5 Department of Obstetrics and Gynecology, University Hospital Ferrara, Ferrara, Italy; 6 Department of Obstetrics and Gynecology, University Hospitals Leuven, 3000 Louvain, Belgium; 7 Clinic of Obstetrics and Gynecology, Santa Maria Della Misericordia University Hospital, Azienda Sanitaria Universitaria Friuli Centrale, Udine, Italy; 8 Department of Medicine, University of Udine, Udine, Italy

**Keywords:** Pressurized intraperitoneal aerosol chemotherapy, PIPAC, Ovarian cancer, Peritoneal carcinomatosis

## Abstract

**Background:**

PIPAC is a recent approach for intraperitoneal chemotherapy with promising results for patients with peritoneal carcinomatosis. A systematic review was conducted to assess current evidence on the efficacy and outcomes of PIPAC in patients affected by ovarian cancer.

**Methods:**

The study adhered to the PRISMA guidelines. PubMed, Google Scholar and ClinicalTrials.gov were searched up to December 2023. Studies reporting data on patients with OC treated with PIPAC were included in the qualitative analysis.

**Results:**

Twenty-one studies and six clinical trials with 932 patients who underwent PIPAC treatment were identified. The reported first access failure was 4.9%. 89.8% of patients underwent one, 60.7% two and 40% received three or more PIPAC cycles. Pathological tumour response was objectivated in 13 studies. Intra-operative complications were reported in 11% of women and post-operative events in 11.5% with a 0.82% of procedure-related mortality. Quality of life scores have been consistently stable or improved during the treatment time. The percentage of OC patients who became amenable for cytoreductive surgery due to the good response after PIPAC treatment for palliative purposes is reported to be 2.3%.

**Conclusion:**

The results showed that PIPAC is safe and effective for palliative purposes, with a good pathological tumour response and quality of life. Future prospective studies would be needed to explore the role of this treatment in different stages of the disease, investigating a paradigm shift towards the use of PIPAC with curative intent for women who are not eligible for primary cytoreductive surgery.

**Graphical abstract:**

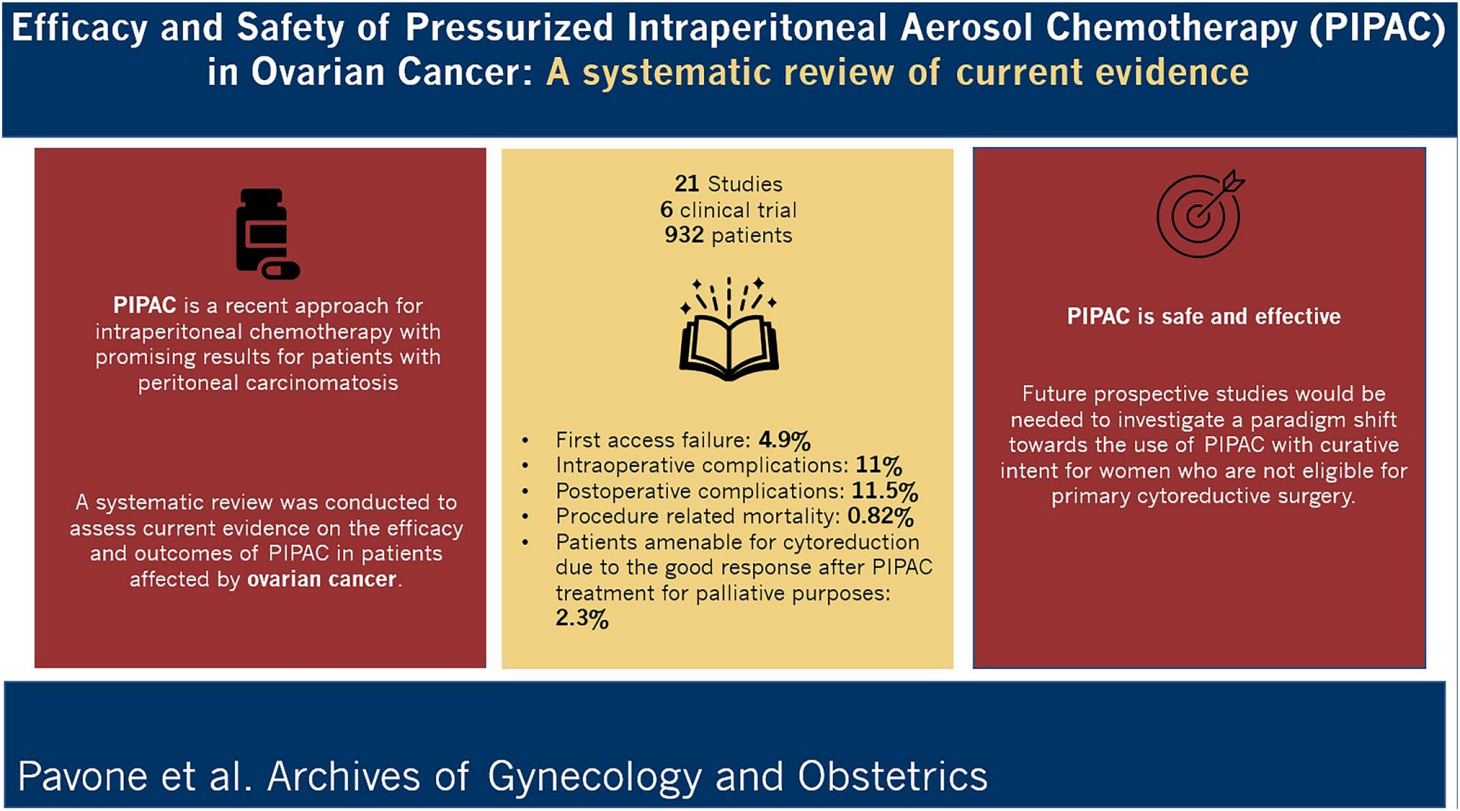

## Introduction

Ovarian cancer (OC) is the second most common and the leading cause of death among gynecological cancers in high-resource countries [1]. Over three-quarters of patients are found to have an advanced stage when first diagnosed, with a disease spreading beyond the ovaries and peritoneal carcinomatosis (PC). Primary debulking surgery (PDS) followed by systemic therapy and maintenance drugs is to date the first choice in patients with a primary diagnosis who are candidates for surgery [2]. Neoadjuvant therapy followed by interval debulking surgery (IDS) is the option if the PDS is supposed to be not feasible according to Fagotti’s score [3], while patients who are not eligible for cytoreduction (disease extension or comorbidities) could directly undergo chemotherapy strategies [2]. In the case of recurrent ovarian cancer (ROC) with PC, the standard approach is systemic chemotherapy, with the chance of targeted maintenance treatment using PARP inhibitors in platino-sensitive selected patients [4]. Moreover, the AGO DESKTOP III/ENGOT ov20 trial has shown that a specific group of patients with ROC can benefit from secondary cytoreductive (SCS) surgery followed by systemic chemotherapy [5]. However, for the majority of patients who experience disease progression after initial or subsequent recurrences, palliative systemic chemotherapy remains the recommended course of action [6]. Systemic chemotherapy has demonstrated effectiveness in treating parenchymal metastases, but its efficacy is notably diminished when it comes to PC also when deriving from different origins (among others: gastric, bowel, appendix, pseudomyxoma, mesothelioma). In recent years to overcome the limitation of poor PC response related to low drug uptake, loco-regional intraperitoneal chemotherapy options have been developed [7, 8]. Hyperthermic intraperitoneal chemotherapy (HIPEC) is a therapeutic approach where heated chemotherapy, which enhances the penetration of drugs in tissues, is directly delivered into the peritoneal cavity after cytoreductive surgery [9].

The results of the randomized OVHIPEC study support the effectiveness of incorporating (HIPEC) into IDS for patients with stage III ovarian cancer demonstrating a 10% survival advantage over a 5-year period [7, 10]. Clinical trials are underway to investigate the integration of the treatment at the time of primary cytoreductive surgery PDS or the HIPEC repetition in the event of relapses [11, 12].

Pressurized intraperitoneal aerosol chemotherapy (PIPAC), was first used on humans in 2011, is an innovative and minimally invasive approach that allows delivering chemotherapy by pressurized aerosol allowing a more homogenous drug distribution and a deeper tissue penetration than peritoneal lavage [13, 14].

Initial evidence for the effectiveness of PIPAC has shown promising results, demonstrating tumor regression in cases where systemic chemotherapy had proven ineffective [15], low toxicity [16] and improved median survival rates [17]. The aim of this systematic review is to evaluate the current evidence on the efficacy and outcomes of PIPAC in patients affected by ovarian cancer.

## Materials and methods

### Search strategy and data extraction

The systematic review was conducted according to Preferred Reporting Items for Systematic Reviews and Meta-Analyses (PRISMA) guidelines [18]. Before data extraction, the review was registered with the International Prospective Register of Systematic Reviews PROSPERO (Registration No CRD42023433670).

The studies included for analysis were obtained by querying the PubMed database, Google Scholar and Clinicaltrial.gov filtered by the English language. No additional filters were applied to the search strategy that started in April 2023 and was completed in December 2023.

The keywords used were “pressurized”, “intraperitoneal”, “aereosol”, “chemoterapy”, “PIPAC”, “ovarian”, “cancer”.

After removing duplicate publications at the title/abstract level, MP and FJ independently reviewed titles, abstracts, and keywords for first-selection purpose. Relevant sources and online links were manually searched, and cross-referencing was conducted for the chosen articles. In case of differences in the selection, the final decision was taken through a discussion with a third author (CT).

In all articles potentially suitable for the purposes of this analysis, the full text was examined independently by MP and FJ in the event of discrepancies, we proceeded as described above.

**Fig. 1:**
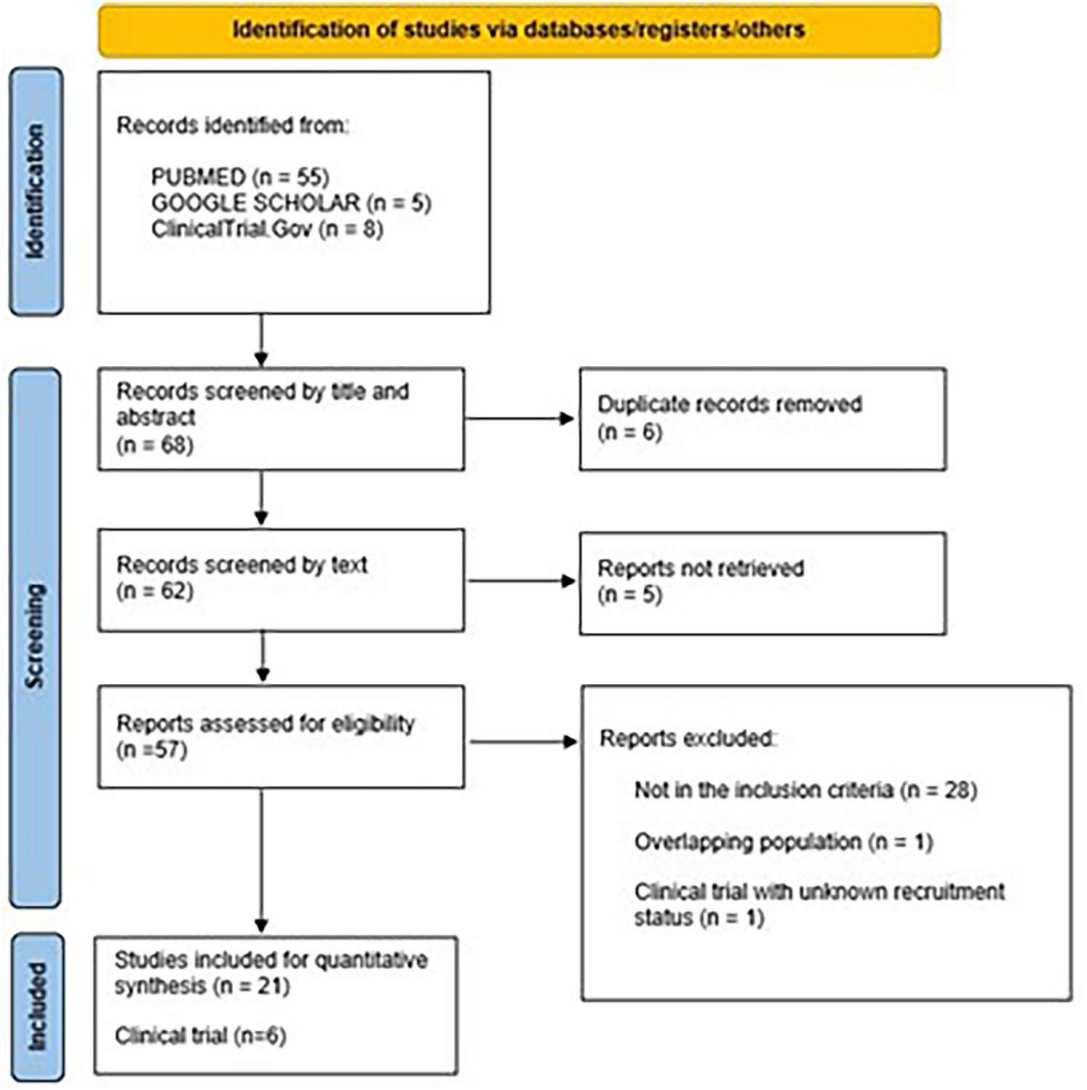
PRISMA flow diagram for studies selection

Studies including patients with OC who underwent PIPAC treatments were selected. Exclusion criteria included duplicate publications, reports about hyperthermic intraperitoneal chemotherapy, non-English language literature, abstracts, letters, editorials, and reviews not reporting original data. Due to our focus on clinical evidence relevant data from the selected studies were independently collected by the reviewers and after common agreement were considered for the systematic review. Figure 1 shows the PRISMA flowchart for studies selection.

### Data analysis

When available the following items were extracted from selected studies: Authors; year of publication; number of patients with OC in the study population; previous chemotherapy treatments; number of PIPAC surgery and details on the procedure (access failure, number of cycles, intra-operative complication, drugs used for the treatment); Postoperative outcomes (complications and toxicity) were graded according to the National Cancer Institute Common Terminology Criteria for Adverse Events 4.0 (CTCAE) [19].

Pathological tumor response was reported, when assessed, according improvement of the peritoneal cancer index (PCI) [20], in alternative the Response Evaluation Criteria in Solid Tumors (RECIST) [21] or the peritoneal regression grading score (PRGS) [22] were considered; Quality of life statements were evaluated and objectivated when EORTC QLQ C-30 [23] or SF-36 [24] questionaries were accessible in the included studies. Decrease in ascites (mL) and subsequent cytoreductive surgery were finally evaluated if indications were available in the included articles.

Meta-analysis was not performed due to the heterogeneity of the original data and outcome measures. Plus, most of the studies included not only OC patients rendering a meta-analysis of the data not statistically significative. Simple statistical evaluations (averages, percentages) were carried out by analyzing the results extrapolated from studies when present and qualitative data synthesis was applied in the outcomes presentation (Tables 1, 2).

**Table 1:** Included studies, PIPAC details

Authors	Study design	Tumor	Sample size	PIPACS	FIRST access failure	Median PCI	> 2 CHT	PIPAC cycle on total			Drugs concentration in OC
								I	II	≥ III	
Tempfer et al. [26]	PCS	OC	21	34	03/34 (9%)	NA	NA	18/21 (86%)	8/21 (38%)	4/21 (19%)	C (7.5), D (1.5)
Solass et al. [13]	CR	OC	1	6	0	14	NA	1 (100%)	1 (100%)	1 (100%)	C (7.5), D (1.5)
Tempfer et al. [27]	PHASE II	OC	64	130	11/130 (8.4%)	NA	NA	53/64 (82%)	43/64 (67.1%)	34/64 (53.1%)	C (7.5), D (1.5)
Giger-Pabst et al. [29]	CR	OC	1	8	0	NA	NA	1 (100%)	1 (100%)	1 (100%)	C (7.5), D (1.5)
Tempfer et al. 2017 [28]	CR	OC	1	13	0	25	NA	1 (100%)	1 (100%)	1 (100%)	C (7.5), D (1.5)
Tempfer et al. [30]	PHASE I	OC	15	34	0	16.3 mean	7 (46.6%)	15/15 (100%)	11/15 (73%)	8/15 (53.3%)	C (7.5), D (1.5) C (9.0), D (1.8) C (10.5), D (2.1)
Somashekar et al. [32]	PCS	OC	3	9	0	19.2 mean	NA	3/3 (100%)	3/3 (100%)	3/3 (100%)	C (7.5), D (1.5)
Odendahl et al. [31]	RCS	OC; CR; G; M; O	91 (25 OC)	158	0	16 mean	NA	NA	NA	NA	C (7.5), D (1.5)
Teixeira Farinha et al. [33]	RCS	OC; O	42 (21 OC)	91	0	10 (5–17)	NA	NA	NA	NA	C (7.5), D (1.5)
Robella et al. [34]	RCS	OC; CR; G; O	14 (3 OC)	57	0	17 (12–21)	NA	NA	NA	NA	C (7.5), D (1.5)
Hübner et al. [43]	RCS	OC; CR; G	44 (21 OC)	91	2/91 (2%)	10 (5–17)	13 (31%)	NA	NA	NA	C (7.5), D (1.5)
Alyami et al. [35]	RCS	OC; CR; G; M; PMP; O	73 (13 OC)	164	0	19 (1–39)	44 (60.2%)	45/73 (61%)	31/73 (42.4%)	8 (11%)	C (7.5), D (1.5)
Kurtz et al. [36]	RCS	OC; CR; G; PMP; O	71 (6 OC)	131	8/131 (6.1%)	19.3 mean	17 (23.9%)	63/71 (88.7%)	40/71 (56.3%)	20/71 (28%)	C (7.5), D (1.5)
Larbre et al. [37]	RCS	OC; G; O	43 (11 OC)	175	0	17 (5–39)	23 (53.5%)	43/43 (100%)	43/43 (100%)	43/43 (100%)	C (7.5), D (1.5)
Siebert et al. [38]	RCS	OC; CR; O	26 (3 OC) Bevacizumab	88	0	20 (8–39)	NA	NA	NA	NA	C (7.5), D (1.5)
		OC; CR; G; O	108 (18 OC) non-Bevacizumab	309	0	16 (0–39)	NA	NA	NA	NA	C (7.5), D (1.5)
Sgarbura et al. [39]	RCS	OC; CR; G; M; O	101 (5 OC)	251	0	19	NA	101/101 (100%)	65/101 (64.3%)	48/101 (47.5%)	O (92)
Ceribelli et al. [40]	RCS	OC; CR; G; M; PMP; O;	43 (18 OC)	71	5/71 (7%)	NA	13 (30.2%)	38/43 (88.3%)	21/43 (48%)	11/43 (25%)	C (7.5), D (1.5)
De Simone et al. [41]	RCS	OC; CR; G; M; PMP; O	65 (18 OC)	123	2/123 (1.1%)	NA	NA	NA	NA	NA	NA
Račkauskas et al. [42]	RCS	OC; G	15 (6 OC)	34	0	8 (4–15)	2 (13.3%)	15/15 (100%)	10/15 (66.6%)	8/15 (53.3%)	C (7.5), D (1.5)
Mehta 2022 et al. [44]	RCS	OC; CR; G; M; O	47 (15 OC)	82	0	24 (12–39)	10 (21.2%)	47/47 (100%)	21/47 (44%)	7/47 (14.8%)	P (20) C(15), A(4), Do(20) Do(20), O(90), A(4)
Vizzielli et al. [45]	PHASE II	OC	43	98	3/98 (3%)	10 (8–12)*	43 (100%)	43/43 (100%)	30/43 (69%)	21/43 (48%)	C (10.5), D (2.1)

*PCS* prospective case series, *CR* case report, *RCS* retrospective case series, *OC* ovarian cancer, *CR* colo-rectal cancer, *G* gastric cancer, *O* Others, *M* Mesothelioma, *PMP* pseudomyxoma peritonei, *PCI* peritoneal cancer index expressed as median and range if not otherwise specified, *CHT* chemotherapy, *C* cisplatin, *D* doxorubicin, *O* Oxaliplatin, *P* paclitaxel, *A* adriamicin, *Do* docetaxel

*Extension of the disease evaluated by the Fagotti’s score

**Table 2:** Included studies, pathological response, outcomes, and quality of life (QoL)

Authors	Pathological response	IOC	CTCAE grade					PIPAC mortality	CRS and HIPEC after PIPAC	QoL	Decrease of ascites
			1	2	3	4	5				
Tempfer et al. [26]	1CR; 2PR; 3SD	0	12	0	3	2	0	0	0	NA	+
Solass et al. [13]	1 CR	0	NA	NA	NA	NA	NA	0	1	+	NA
Tempfer et al. [27]	26/34** OTR	0	71	32	8	0	0	0	NA	+	NA
Giger-Pabst et al. [29]	1 CR	0	1	1	0	0	0	0	0	+	NA
Tempfer et al. [28]	1 OTR*	0	2	0	2	0	0	0	0	=	NA
Tempfer et al. [30]	2CR; 5PR; 1SD	1 (6.6%)	85		1	0	0	0	0	NA	NA
Somashekar et al. [32]	2 PR; 1SD	0	0	2	0	0	0	0	0	NA	NA
Odendahl et al. [31]	NA	9 (10%)	0	0	8	1	2	2 (2.2%)	NA	=	NA
Teixeira Farinha et al. [33]	NA	4 (9%)	NA	NA	NA	NA	NA	NA	NA	=	NA
Robella et al. [34]	NA	0	6	8	0	0	0	0	NA	=	NA
Hübner et al. [44]	NA	4 (9%)	1	6	0	0	1	0	NA	NA	NA
Alyami et al. [35]	32 OTR*	16 (21%)	0	0	16	0	5	5 (6.8%)	8 (11%)	=	+
Kurtz et al. [36]	10 PRGS1	4 (2.8%)	0	1	1	0	1	0	0	=	+
Larbre et al. [37]	NA	NA	NA	NA	NA	NA	NA	NA	NA	NA	NA
Siebert et al. [38]	NA	13 (15%)	NA	NA	4 (4.5%)			0	NA	NA	NA
	NA	29 (9.4%)	NA	NA	10 (3.2%)			0	NA	NA	NA
Sgarbura et al. [39]	NA	NA	26	4	14	3	1	0	6 (5.9%)	NA	NA
Ceribelli et al. [40]	9 PRGS1; 5 PRGS2	1 (2.6%)	0	0	0	1	0	0	3 (6.9%)	NA	NA
De Simone et al. [41]	6 PRGS1; 1 PRGS2; 16 PRGS3; 6 PRGS4	0	0	0	2	1	0	0	0	=	NA
Račkauskas et al. [42]	NA	0	0	1	1	1	0	0	0	NA	+
Mehta et al. [44]	4 PRGS1-2	2 (4.2%)	24 (51%)		6 (12.7%)		0	0	8 (17%)	NA	+
Vizzielli et al. [45]	8 PRGS3; 19 PRGS4	2 (4.6%)	NA	6 (13.9%)	2 (4.6%)	0	0	0	NA	+	NA

*CR* complete response, *PR* partial response, *SD* stable disease, *PRGS* peritoneal regression grading score

^a^*OTR* objective tumor response (tumor regression on histology, stable disease on repeated video-laparoscopy and peritoneal carcinomatosis index)

^b^Considered patients after 3 PIPACs

### Procedure

In the selected studies, the surgical procedure was performed according to standard. After induction of the pneumoperitoneum at 12 mmHg CO2, two 5-and 12-mm balloon trocars are inserted into the abdominal wall. The PCI is evaluated based on lesions distribution. Peritoneal biopsies are performed for histological confirmation or assessment of tumour pathological response. The volume of ascites is recorded, then the fluid aspirated. A nebuliser (until 2015 MicroPump, Reger, Villingendorf, Germany; since 2015 CapnoPen, Capnomed, Villingendorf, Germany) is connected to a high-pressure intravenous injector (Injektron 82 M, MedTron, Saarbruecken, Germany) and inserted into the abdomen. A pressurized aerosol containing the chosen drug doses is applied via nebulizer and injector. The flow rate is usually 30 mL/min with a maximum upstream pressure of 200 psi in the high-pressure injector. The injection is monitored remotely to exclude occupational exposure. The therapeutic capnoperitoneum is held for 30 min at a temperature of 37 °.

## Results

A search strategy was developed and applied to PubMed, Google Scholars and ClinicalTrial.Gov databases where sixty-eight citations were yielded. Once duplicates were removed, sixty-two studies were further investigated for inclusion. One retrospective study was excluded for overlapping populations with the other two prospective studies [25]; One clinical trial was removed for the unknown status of the recruitment and twelve studies not selected for not meeting the inclusion criteria. No additional studies were found through the reference lists of included ones or from relevant systematic reviews. Twenty-one studies [11, 13, 26–45] and six clinical trials were finally evaluated for the qualitative synthesis of results as shown in Fig. 1.

There were 932 patients in total who underwent PIPAC treatment. Of these 332 were affected by OC. Six studies addressed only women with OC (149 patients) [22, 26–29, 45] while the others included also patients with PC from other origins (colon, gastric, breast and peritoneal cancers). In total 2305 PIPAC procedures were performed in the analysed studies. The individual results presented are a synthesis of the data extrapolated from the single articles as shown in Tables 1, 2. The reported first access failure was of 4.9% and the median PCI before the treatment varied among the studies from 10 to 24. Nine studies [30, 35–37, 40, 42–45] reported data on previous chemotherapy treatment in a population of 351 patients, of these 43.6% underwent > 2 cycles of systemic therapy before the PIPAC surgery. Number of PIPACs cycles were reported in fifteen [13, 26–30, 32, 35–37, 39, 40, 42, 44, 45], of 542 patients 89.8% underwent one, 60.7% two and 40% received three or more PIPAC cycles. The majority of patients with OC received PIPAC with cisplatin 7.5 mg/m^2^ and doxorubicin 1.5 mg/m^2^ while the dose was escalated up to cisplatin 10.5 mg/m^2^ and doxorubicin 2.1 mg/m^2^ in the series of Tempfer et al. [30]. In one study patients receive oxaliplatin 92 mg/m^2^ [39] while Mehta et al. proposed a regimen of docetaxel 20 mg/m^2^—cisplatin mg/m^2^—Adriamycin 4 mg/m^2^ or docetaxel 20 mg/m^2^—oxaliplatin 90 mg/m^2^—adriamycin 4 mg/m^2^ or paclitaxel 20 mg/m^2^ alone [44]. The efficacy of the treatment was confirmed by studies reporting objectively pathological tumor response as shown in Table 2.

The feasibility, safety, and tolerance of repeated treatment with PIPAC were demonstrated by the presence of a low rate of intraoperative complications (11%), 97 adverse post-operative events (11.5%) reported as CTCAE ≥ 3 on 846 patients and the 0.82% of procedure-related mortality. Quality of life scores have been consistently stable or improved during the treatment time and four studies reported a decrease in ascites volume during treatment with symptom relief. Finally, the percentage of patients undergoing CRS (cytoreductive surgery) with or without HIPEC after PIPAC treatment for palliative purposes is reported to be 2.3% in studies considering only OC patients and it rises to 5.6% if the total of studies is considered. Several clinical trials are currently ongoing with the aim of testing new pharmacological dosages, innovative drugs combination, toxicity and clinic-pathological response rates (Table 3).

**Table 3:** Ongoing clinical trials on PIPAC in OC patients

Registry number	Sample	Country	Study phase	Intervention	Primary endopoint	Recruitment status
NCT04811703	15	France	I	Addition of cisplatin-doxorubicin PIPAC sessions to carboplatin-paclitaxel systemic chemotherapy	Dose-limiting toxicities	RECRUITING
NCT01809379	69	Germany	II	Chemotherapy with doxorubicin and cisplatin	Clinical Benefit Rate (CBR) according to RECIST criteria	COMPLETED
NCT03304210	20	Belgium	I	PIPAC with Abraxane	Maximally tolerated dose (MTD) of Abraxane	COMPLETED
NCT04329494	49	USA	I	PIPAC with cisplatin, doxorubicin, oxaliplatin, leucovorin, fluorouracil, mitomycin, and irinotecan	Dose limiting toxicities	RECRUITING
NCT04000906	36	Switzerland	I	Combination of nab-paclitaxel and cisplatin	Determine the maximal tolerated dose (MTD) of Nab paclitaxel (Abraxane®) administered IP by PIPAC in concomitance with cisplatin	RECRUITING
NCT02604784	105	Italy	I-II	Overall Response Rate (ORR) of oxaliplatin, or cisplatin and doxorubicin	Overall Response Rate (ORR) according to RECIST criteria (version 1.1) after 2 and 3 cycles of PIPAC	COMPLETED

## Discussion

Peritoneal carcinomatosis is a frequent outcome of ovarian cancer. Chemotherapy with pressurised intraperitoneal aerosol is a recently introduced and minimally invasive technique that has gained attention as a treatment option for OC patients with PC when systemic or surgical therapy is not possible [46]. To date, there is no evidence of the PIPAC effectiveness over systemic therapy, and this treatment is only adopted for palliative purposes.

Based on 2305 procedures in 932 patients, studies on the role of PIPAC in ovarian cancer have shown promising results. PIPAC can deliver high concentrations of chemotherapeutic agents directly to the peritoneal surface, increasing local drug concentration and enhancing tumour response. It has been observed to induce tumour regression and improve the quality of life of patients with a low rate of complications and procedure-related mortality. For patients with OC and PC, systemic therapy is almost always ineffective due to the difficulty of drug uptake by the peritoneal lesions [47]. Multiple lines of chemotherapy then, in addition to the gradual lack of efficacy, lead to a decline in quality of life due to drug toxicity [2]. One of the major problems leading to the exclusion of 5% of possible candidates is the inability to access the abdominal cavity because of adhesions from previous surgery or the extent of disease. Treatments with PIPAC have proven effective in inducing an objectively pathological tumour response in these patients, especially when at least two cycles have been carried out [27]. The pharmacological standard represented by cisplatin 7.5 mg/m^2^ and doxorubicin 1.5 mg/m^2^ was thoroughly investigated. However, the Tempfer et al. dose escalation study showed good tolerability even at doses of cisplatin 10.5 mg/m^2^ and doxorubicin 2.1 mg/m^2^ [30]. Oxaliplatin commonly administered for gastrointestinal tumours has shown efficacy also when applied to OC although phase II and III studies are still needed to confirm its efficacy [39]. The supplement of bevacizumab to PIPAC has also been described as viable by Siebert et al., highlighting several possible undiscovered paths to further investigate [38]. Different drug combinations have been demonstrated to be safe for these patients, and clinical trials are underway to better define the maximum dosage of cisplatin and doxorubicin (NCT04811703) to test the introduction of (NCT03304210) or other pharmacological formulations (NCT02604784). In patients with the treatment-refractory disease, good quality of life is a priority in the choice of treatment choice. When assessing the clinical significance of a new treatment for palliative care, it is crucial to consider the tolerability of treatment-related side effects. If a therapy is less toxic compared to existing treatments, even a modest improvement in efficacy can be considered acceptable. Conversely, if a therapy carries significant toxicity, it should be justified by a substantially greater expected benefit to achieve a meaningful clinical outcome. The largest prospective phase II study (PARROT Trial) [45] in women with platinum-resistant recurrent ovarian cancer was recently published. In this study, in 82% of women, a clinical benefit rate was achieved, further confirming that PIPAC is a feasible approach for these patients without impacting on quality of life. From the results of this systematic review, the QoL objectified in the studies with validated questionnaires did not worsen during treatment and instead improved or remained stable with an advance in symptoms related to an objective reduction in ascites volume. Odendahl et al. evaluated QoL in 91 patients with advanced PC. They found that QoL was preserved, the gastrointestinal symptoms did not recover during PIPAC therapy, and the pain score was improved in 32% of patients [31]. The same enhancement was reported by a phase two study [25] and confirmed by Teixeira Farinha et al. stating that PIPAC as treatment of PC had no negative impact on patients’ overall QoL [33].

Furthermore, the induced organ-specific renal and hepatic toxicity is stated to be acceptable [34, 37, 40] making this treatment an accessible choice also for elderly patients with a good ECOG [29].

In the current literature, mortality associated with PIPAC reached up to 8.3% for patients with PC from other origins than OC [48] and major complications occurred in 0–37% of patients [17]. This high mortality is attributable to disease progression in a population of selected patients who were treated with palliative intent [48]. From the selected articles, however, if only events related to the PIPAC procedure are considered, the mortality rate is extremely low (0.82%), and major complications occurred in 11.8% of a group of terminal and therefore already frail patients. Moreover, the median survival after PIPAC is reported to be 11–14.1 months for patients with OC [17]. This survival is about average compared to what has been reported in the literature for other tumour types. For instance, Alyami et al. [49] shared findings from the Lyon cohort during the 38th European Society of Surgical Oncology meeting. They reported a median survival of 19.1 months for patients with gastric peritoneal metastasis. These initial outcomes are encouraging when contrasted with one of patients who solely received systematic chemotherapy, where the median survival did not surpass 10.7 months (95% CI 9.1–12.8) [50]. For patients with PC from malignant mesothelioma and colon cancer, clinical trials are underway to assess long-term survival, whereas after systemic therapy alone it is estimated to be 16.3 and 12 months, respectively [51, 52]. The possibility of taking biopsy samples during PIPAC cycles has made it possible to obtain objective data on the histological response of the tumour superior to that assessed by radiological imaging during systemic therapy. Due to this, the effectiveness of this method of delivery has been widely recognized, to the extent that in certain documented instances, patients who were originally considered suitable only for palliative supportive treatment have been able to undergo cytoreductive surgery (2.3% for peritoneal unresectable OC metastasis and 5.6% for others origin), with or without HIPEC, following PIPAC cycles. From this evidence, first described by Girshally et al. [53], it is possible to imagine how, if PIPAC treatment were not reserved only for patients with no other therapeutic options and with already advanced disease, the efficacy of the treatment could be greater. Moreover, there is no available data from randomized phase III trials comparing the efficacy of PIPAC to systemic chemotherapy and to date PIPAC is still only an option in patients after multiple lines of chemotherapy unwilling or unable to undergo further systemic treatments.

It is important to observe the exclusion criteria when considering PIPAC, which include a life expectancy of fewer than three months, intestinal occlusion, exclusive reliance on total parenteral nutrition, severe ascites, and having experienced a previous severe allergic reaction to the chemotherapy drug used. Additionally, there are relative contraindications such as metastases outside the peritoneal cavity, performance status greater than two according to the ECOG (Eastern Cooperative Oncology Group performance status), and the presence of portal vein thrombosis [54]. Typically, a minimum of three PIPAC procedures are performed at intervals of approximately six to eight weeks, but the subsequent treatment can be adjusted based on the patient’s tolerance and response to the therapy. PIPAC can be administered as a standalone treatment or in combination with concurrent systemic therapy. Many centers recommend discontinuing systemic treatment for a period of two weeks before and one week after the PIPAC procedure [34]. This is the first systematic review focusing on all published studies including PIPAC in women with OC conducted according to PRISMA guidelines [18]. The results showed that treatment with repeated cycles of PIPAC is safe and effective for palliative purposes, with a good pathological tumour response and quality of life. However, at this point in time, no evidence supporting the use of PIPAC in other settings is available and prospective studies would be needed to investigate the role of this treatment in different stages of the disease, proposing a paradigm shift towards the use of PIPAC with curative intent for women who are not eligible for PDS or HIPEC.

## Data Availability

All data generated or analyzed in this review are included in this article and/or its figures. Further enquiries can be directed to the corresponding author.
